# Batik marker as an implementation of prevention of contact transmission of MDRO patients

**DOI:** 10.1017/ash.2025.97

**Published:** 2025-09-03

**Authors:** Puspa Wardhani, Musofa Rusli, Nita D. Ali, Tutuk Nurwahyuni, Bela Y. Selfia

**Affiliations:** 1 Department of Clinical Pathology, Faculty of MedicineAirlangga University; 2 Department of Internal Medicine, Faculty of MedicineAirlangga University; 3 PCI Committee, Dr. SOETOMO General Academic Hospital Surabaya

**Keywords:** Antimicrobial Resistance Patient Marker, Hand Hygiene, Infection Control Practices, Antimicrobial Resistance Patient Supervision

## Abstract

**Background:** WHO estimates over 700,000 deaths globally annually due to antimicrobial resistance. One of the main factors of antimicrobial resistance is non-compliance with infection control measures. Contact alert markers must be installed in the patient area to remind officers to carry out infection transmission prevention to prevent the failure of infection control practices. The provision of red markers and information on the infection suffered by patients creates a stigma that is less accepted because of patient privacy. This study aimed to evaluate the modified marker with a batik design as local wisdom without infection information, as a reminder of infection control practices by officers, and to maintain patient privacy. **Method:** The Committee for Infection Prevention and Control modified the design of markers of patients with antimicrobial resistance. Batik markers were designed with hand hygiene reminders and batik motifs as local wisdom so as not to cause negative stigma. Batik markers were implemented in the patient’s bed area so that it was easy for officers to understand how to implement infection control practices and supervise patients with antimicrobial resistance. **Results:** Modifying the marker design was more acceptable to the patient’s family than the previous one. Adherence to supervision filling of antimicrobial resistance patients was performed in all patients. Adherence to Hand Hygiene increased by up to 4% in one month. With the Batik Marker, officers could easily recognize the marker so that infection control practices could be carried out according to hospital regulations. **Conclusion:** Antimicrobial resistance is an increasing health threat. A type A hospital requires an intradisciplinary approach and collaborative efforts to prevent and control it. Implementing Batik Markers at inpatient areas with antimicrobial resistance makes it easier for staff to implement increased contact awareness, supervision recording, and improved hand hygiene without causing rejection from the patient’s family.

